# ﻿A new species of *Silvatares* (Trichoptera, Pisuliidae) from the Democratic Republic of the Congo

**DOI:** 10.3897/zookeys.1111.85307

**Published:** 2022-07-11

**Authors:** Ernesto Rázuri-Gonzales, M. François Ngera, Steffen U. Pauls

**Affiliations:** 1 Department of Terrestrial Zoology, Entomology III, Senckenberg Research Institute and Natural History Museum, Frankfurt, Germany Senckenberg Research Institute and Natural History Museum Frankfurt Germany; 2 Loewe Centre for Translational Biodiversity Genomics (LOEWE-TBG), Frankfurt, Germany Loewe Centre for Translational Biodiversity Genomics Frankfurt Germany; 3 Centre de Recherche en Sciences Naturelles, Lwiro, Bukavu, Democratic Republic of the Congo Centre de Recherche en Sciences Naturelles Lwiro Democratic Republic of the Congo; 4 Institute of Insect Biotechnology, Justus-Liebig University, Gießen, Germany Justus-Liebig University Gießen Germany

**Keywords:** Africa, new species, taxonomy, Trichoptera

## Abstract

A new species of caddisfly in the family Pisuliidae from the Democratic Republic of the Congo is described and illustrated herein, *Silvataresholzenthali***sp. nov.** Based on the presence of a pair of spines on the endotheca, this species belongs to the *thrymmifer* group. Additionally, *Silvatareslaetae* is recorded for the first time from the D.R. Congo.

## ﻿Introduction

The caddisfly genus *Silvatares* Navás, 1931, along with *Pisulia* Marlier, 1943, belong to the African endemic family Pisuliidae. The species currently placed in *Silvatares* were originally included in the genus *Dyschimus* Barnard, 1934. Stoltze (1989) subsequently reviewed the family and included nine species in *Dyschimus*. Later, Prather and Holzenthal (2002) synonymized *Silvatares* and *Dyschimus*, thereby transferring all the species in the latter genus to *Silvatares* on the grounds that this name had precedence. More recently, Ngirinshuti et al. (2019) described an additional species from Rwanda, raising the number of hitherto known species to eleven.

Species of *Silvatares* generally inhabit forested streams in sub-Saharan Africa (Table 1). The genus *Silvatares* is characterized in the adult stage by their larger size (vs. *Pisulia*), the shape of their maxillary palps, and a tibial spur formula of 2-4-4. The larvae are also large, have two or three accessory hooks on the anal claws, and the dorsal hump is absent on the first abdominal segment. A peculiarity of all Pisuliidae larvae are the cases. These are constructed from plant materials and are triangular in cross-section (Stoltze 1989; SUP, FNM, pers. obs.).

**Table 1.** Species of *Silvatares*, with their known distributions and life stages.

Species	Distribution	Known life stages
*Silvatareschitae* (Stoltze, 1989)	Tanzania	male, female
*Silvatarescollyrifer* (Barnard, 1934)	South Africa	male, larva, pupa
*Silvatarescrassus* (Stoltze, 1989)	Tanzania	male, female
*Silvataresensifer* (Barnard, 1934)	South Africa	male, female
*Silvataresexcelsus* Navás, 1931	Uganda, DRC	male
*Silvataresfurcifer* (Marlier, 1953)	DRC	female, larva, pupa
*Silvataresholzenthali* sp. nov.	DRC	male
*Silvatareslaetae* Ngirinshuti & Johanson, 2019	Rwanda, DRC*	male
*Silvatareslonginquus* (Gibbs, 1973)	Ghana	male, female, larva**
*Silvataresmadagascariensis* (Stoltze, 1989)	Madagascar	male
*Silvataresornithocephalus* (Stoltze, 1989)	South Africa	male
*Silvataresthrymmifer* (Barnard, 1934)	South Africa	male, female, larva, pupa

* new country record. ** Gibbs did not formally describe the larva of *S.longinquus* but compared its appearance to *S.furcifer*.

Stoltze (1989) informally subdivided the genus into three species groups: the *madagascariensis* group, characterized by the large internal lobes on the male tergum IX (including *S.madagascariensis*); the *ensifer* group, characterized by the presence of large lateral processes from the phallobase (including *S.collyrifer*, *S.ensifer*, *S.longinquus*, and *S.ornithocephalus*); and the *thrymmifer* group, characterized by the presence of a pair of apical spines on the endotheca (including *S.chitae*, *S.crassus*, *S.excelsus*, *S.furcifer*, *S.laetae*, and *S.thrymmifer*).

While identifying caddisfly material from our current survey of the fauna of the Democratic Republic of the Congo, we discovered a new country record and a new species of *Silvatares*. Herein we describe and illustrate this new species, based on a single male specimen.

## ﻿Materials and methods

### ﻿Study area

The Kahuzi-Biega National Park (KBNP: 1°36'S to 2°37'S, 27°33'E to 28°46'E) is a UNESCO World Heritage Site. It is located 20 km west of Bukavu, South Kivu Province, in the Democratic Republic of the Congo. The park was created in 1970 with 600 km^2^ and was later extended to 6,000 km^2^ in 1975 (Ngera et al. 2019). The park includes lowland and highland areas connected by a 7.4 km wide by 20 km long corridor. The KBNP is considered a biodiversity hotspot with a high rate of endemic species. Most studies carried out in the park have focused on mammals, birds, plants, and reptiles. Insects have rarely been studied in the area (Ngera et al. 2019).

The eastern part of the KBNP consists of high-elevation zones, ranging from 1800 m to 3308 m a.s.l. Bamboo forests, primary and secondary mountain forests, and swamp forests are the most common vegetation types in this area. Aquatic ecosystems include rivers, streams, and wetlands. The rivers and streams of the western flank of these mountains drain into the Lohoho and Luha rivers, both supplying water to one of the most important tributaries of the Congo River, the Lowa River (Ngera et al. 2019). Rivers and streams draining the eastern flanks flow into Lake Kivu, which is connected to Lake Tanganyika by the Ruzizi River. The soil is mostly of volcanic origin. Mean temperatures vary between 10.0 °C and 18.8 °C.

### ﻿Sampling site

The Lwiro River is located in the northeastern part of the KBNP. It originates in the Cigali swamp on the Kahuzi mountain, flows across a vast high-altitude forest (Tshibati) up to the border of the park. From there it flows through cultivated areas past several villages before draining into Lake Kivu. Within the park, it receives few first-order tributaries. Downstream of the park, several second-order streams flow into the Lwiro. The sampling site (Kakezi) is at 2,120 m a.s.l., ~ 2 km upstream of the Tshibati waterfall, and is dominated by natural forests. The water current averages 56.0 cm/s across lotic and lentic zones. The river is ~ 9.5 m wide with an average depth of ~ 40cm at the time of collection. Rocky substrates (boulders, stones, cobbles) dominate the riverbed, but organic substrates, especially logs and leaf packs also provide important habitat. Physical and chemical parameters showed slightly basic pH throughout the day (7.73 at 06:00 am to 7.8 at noon). Water temperature also increased during the day, measuring from 13.2 °C at 06:00 am to 15.0 °C at noon. Conductivity ranged from 60 to 62 µS/cm, total dissolved solids from 30 to 31 ppm, and dissolved oxygen was relatively low (5.1 mg/L, ~ 50% saturation).

The specimen of *Silvatareslaetae* was collected from the vegetation at the Chashoga swamp (Tshibati sector, Kahuzi-Biega National Park) using a hand net. The elevation for this site is slightly lower than the other site (2,030 m a.s.l.).

### ﻿Morphological methods

The specimen of *S.holzenthali* sp. nov. was collected using a UV light trap and fixed in 96% ethyl alcohol. Specimen preparation and observation was done following standard methods outlined in Blahnik and Holzenthal (2004). The male genitalia were prepared using 80% lactic acid at 90 °C for 1 h. The specimen was examined on an Olympus SZX10 stereoscope, and pencil sketches were made using a drawing tube attached to a Leitz Dialux 20 compound microscope. The pencil sketches were then scanned using a Konica Minolta bizhub C368 multifunction printer and imported into Adobe Illustrator CS6 to serve as a template for the digital illustration.

**Figure 1.**
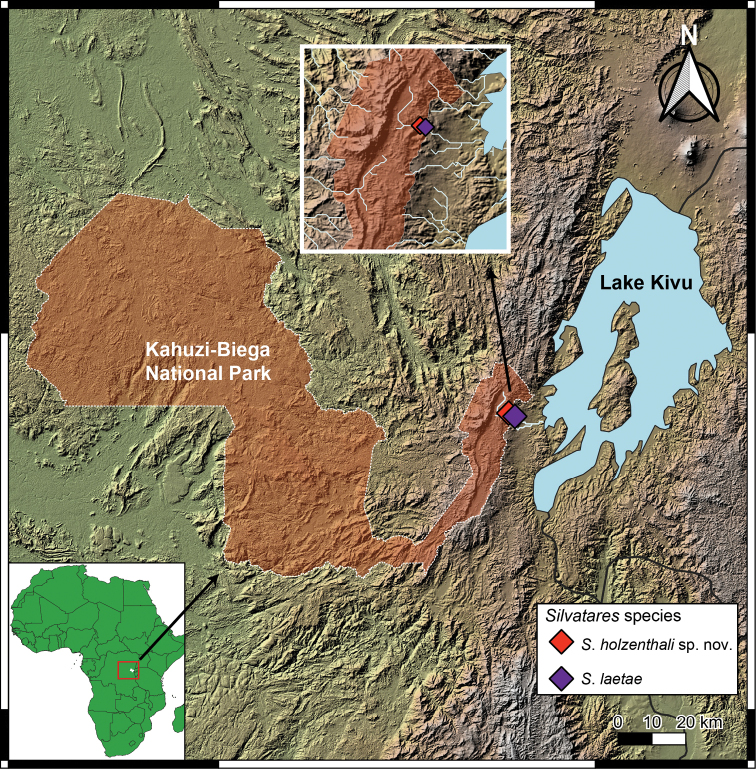
Distribution map of the *Silvatares* species treated in this paper.

The distribution map (Fig. 1) was prepared in QGIS 3.22.4 Białowieża (QGIS Development Team, 2022). Vector and raster maps were prepared with Natural Earth (2018) and CIAT-CSI SRTM (Jarvis et al. 2008) data.

All specimens treated in this paper are stored in 96% ethyl alcohol and are deposited in the Senckenberg Research Institute and Natural History Museum, Frankfurt, Germany (**SMF**).

### ﻿Molecular methods

We removed a pair of legs of the new species and incubated the tissues in 60 µl TNES lysis buffer (100 mM Tris-HCl, 25 mM NaCl, 10 mM EDTA, 1% SDS) and 8 µl Proteinase K (20 mg/ml) overnight. For DNA binding and clean-up, we added 75 µl custom speed-bead suspension (Sera-Mag SpeedBeads Carboxylate, hydrophobic, Cytiva; see Rohland and Reich 2012, Genome Res 22: 939–946), incubated for 15 min on a nutating shaker, and washed the beads twice with 75% ethanol after the supernatant had been removed and discarded. The DNA was eluted from the air-dried beads with 1X TE.

DNA sequences were generated for the cytochrome-c-oxidase subunit I barcoding region (COI, 658 bp) using primers LCO1490-L and HCO2198-L (Nelson et al. 2007). Polymerase chain reactions (PCR) were run on a Mastercycler Pro S (Eppendorf, Hamburg, Germany) in reactions containing 1X MyTaq Reaction Buffer, 0.4 µM of each forward and reverse primer, 0.5 U MyTaq DNA Polymerase, 1 µl DNA and nuclease-free water to fill up to a 10 µl total volume. Reaction conditions were 1 min at 95 °C for initial denaturation followed by 35 cycles of 20 s at 95 °C (denaturation), 30 s at 45 °C (annealing) and 30 s at 72 °C (extension). The reaction ended with a final extension for 5 min at 72 °C. PCR products were visualized on agarose gels and purified using a modified ExoSAP protocol with Exonuclease I (20U/µl) and Fast AP Themosensitive Alkaline Phosphatase (1U/µl; both ThermoFisher Scientific, Vilnius, Lithuania). DNA sequences were generated at the Laboratory Centre of the Senckenberg Biodiversity and Climate Research Centre using a 3730XL DNA Analyzer (Applied Biosystems).

The sequences were edited and aligned in Geneious Prime 2022.1 (Biomatters, New Zealand) and uploaded to BOLD Systems under accession number SPAFT001-22.

## ﻿Results

### ﻿ Silvatares holzenthali sp. nov.

Taxon classificationAnimaliaTrichopteraPisuliidae

320DC1CD-E550-5A60-8B8A-2914B05EA256

http://zoobank.org/A8927970-7F34-49E0-B091-D11EC5BA79B4

Figs 1 , 2 , 3

#### Holotype.

Democratic Republic Of The Congo • ♂; Sud-Kivu, Kahuzi-Biega National Park, Tshibati-Kakezi (up waterfalls); 2.21691°S, 28.77328°Е, 2,120 m a.s.l.; 23 Aug. 2017; Mwangi leg (SMF) [SMFTRI00018633].

#### Diagnosis.

*Silvataresholzenthali* sp. nov. is a member of the *thrymmifer* group of Stoltze (1989) due to the presence of a pair of apical spines on the endotheca. The new species is closest to *S.excelsus* and *S.laetae* based on the presence of inferior appendages with a long, secondary basodorsal lobe. The apex of this lobe in *S.holzenthali* is slightly subtriangular in lateral view, while in *S.laetae* and *S.excelsus* it is slightly capitate. Additionally, tergum X in *S.holzenthali* is broad basally and tapers to a digitate apex while in *S.laetae* and *S.excelsus*, tergum X is broad throughout its length.

#### Description.

**Figure 2.**
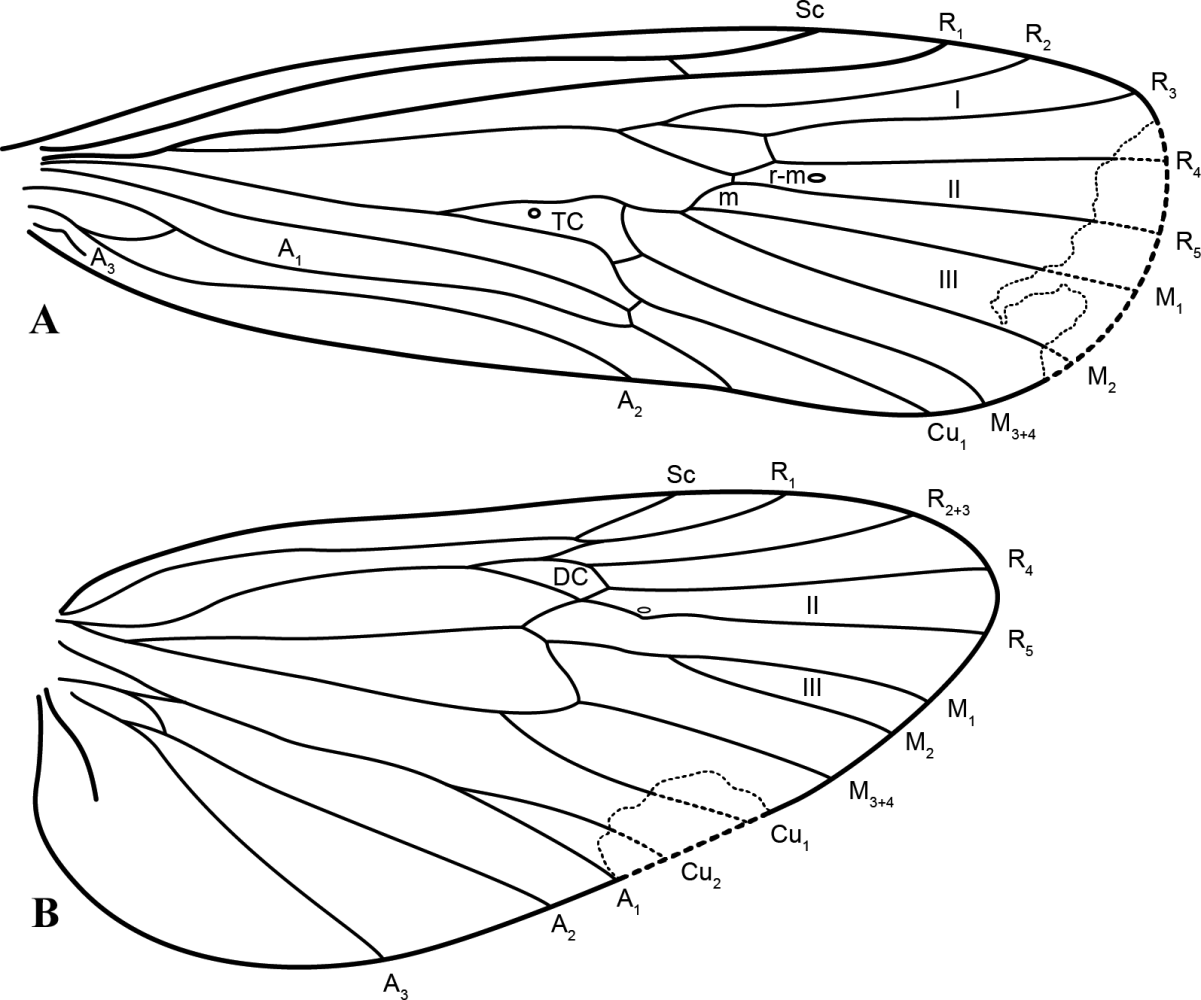
*Silvataresholzenthali*, new species, wing venation **A** forewing **B** hind wing.

**Adult male.** Overall color pale brown (in alcohol). Antennae pale brown with short, whitish setae; antennal segments cylindrical with secondary constriction subapically on each segment; antennae broken. Head and thorax with brown (especially dorsally) and pale brown setae, infraocular wart narrow and long with dark brown setae. Palpi pale brown with brown (especially on apical segment) and pale brown setae. Legs pale brown with short and long dark brown setae. Forewing length ~ 11.7 mm (*n* = 1; forewing apex damaged). Forewing membrane pale brown, except for a whitish mark on apicodorsal corner of thrydial cell, with short brown setae. Forewing (Fig. 2A) with forks I, II, and III present; discoidal cell closed; thrydium present; A_2_ complete, reaching wing margin; A_3_ incomplete and ending before reaching wing margin. Hindwing (Fig. 2B) with forks II and III present; discoidal cell closed; base of Cu_2_ fused to base of A_1_. Segment V with elongate sternal glands, slightly broader apically, globose; segment VII with short ventromesal process.

**Figure 3.**
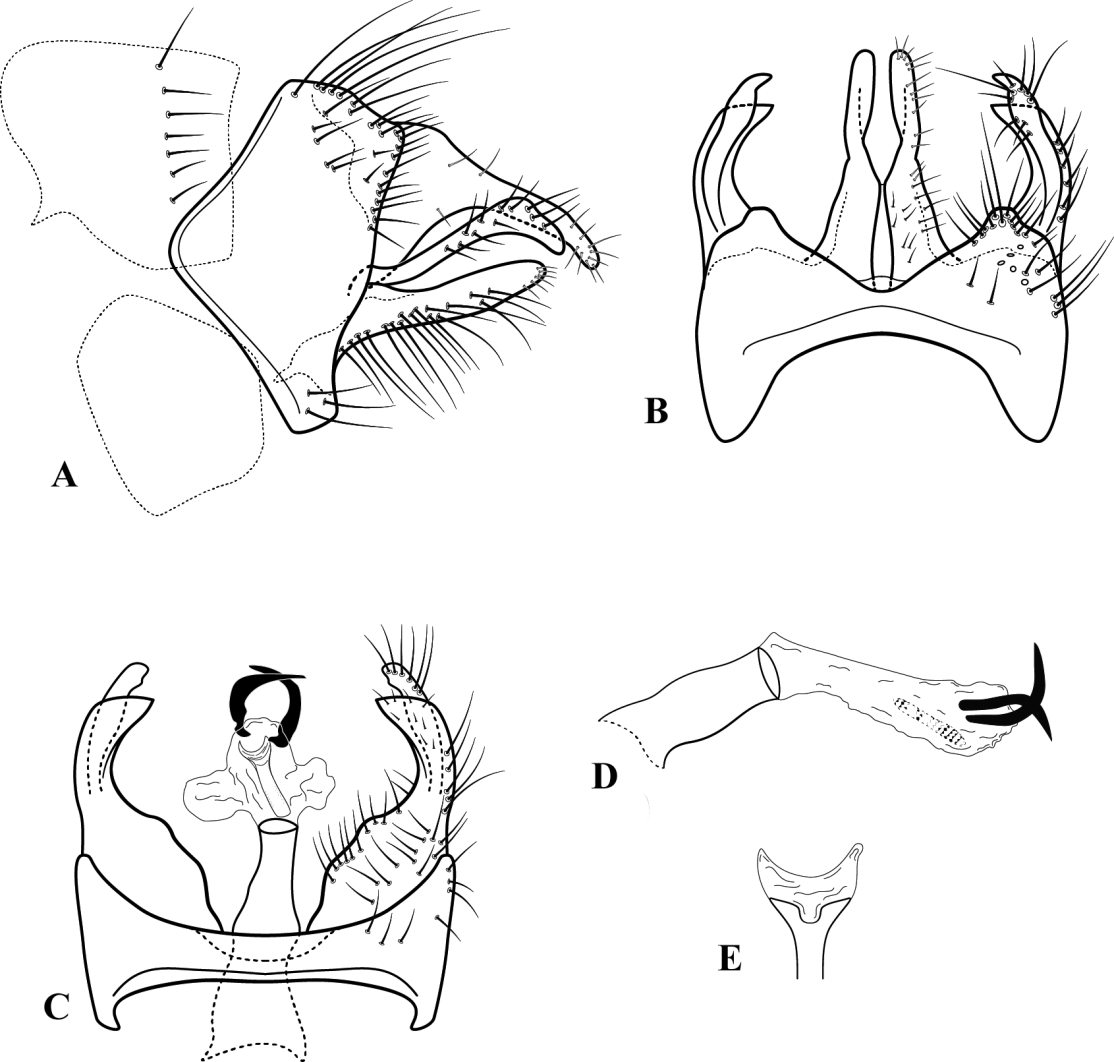
*Silvataresholzenthali*, new species, ♂ genitalia **A** lateral **B** dorsal **C** ventral **D** phallic apparatus, lateral **E** apex of phallic sclerite, dorsal.

***Male genitalia*.** Segment IX (Fig. 3A) in lateral view widest midlaterally, anterior margin produced into broadly rounded lobe, posterior margin very slightly sinuous, dorsal margin longer than ventral margin, setae on ventral and posterodorsal surfaces. Segment IX (Fig. 3B) in dorsal view with posterior margin produced sublaterally and concave mesally, anterior margin broadly concave; in ventral view (Fig. 3C), anterior and posterior margins broadly concave. Tergum X (Fig. 3A) in lateral view broad basally, tapering into digitate process, apex rounded, concave ventrally, down-turned; in dorsal view (Fig. 3B) divided by deep mesal cleft into two setose tergites, setae on lateral and apical margins; mesal margins angulate basally and subapically, lateral margins angulate mesally. Inferior appendages (Fig. 3A) with a dorsal lobe arising basally from dorsal surface. Basal segment long, ventral margin slightly concave basally, rounded apically. Dorsal segment longer than ventral segment, somewhat capitate apically. Phallic apparatus (Fig. 3D) short and stout, endothecal membrane expanded, with a pair of slender, sharply bent, acute endothecal spines apicodorsally, and a slender, Y-shaped sclerite apicoventrally (Fig. 3E; apex of phallic sclerite in dorsal view).

**Female.** Unknown.

**Larva.** Unknown.

#### Etymology.

It is with great pleasure that we name this species after Dr. Ralph W. Holzenthal for his life-long contributions to Trichoptera taxonomy and systematics, especially in the Neotropics. Ralph has not only been an inspiration for Trichoptera researchers across the world but has been a very important mentor for the authors, and most importantly, a very dear friend, encouraging us throughout our careers. We thank Ralph for great craft beer tastings, memorable garden barbecues, fascinating field trips, and woodworking workshops.

#### Distribution.

Democratic Republic of the Congo (Sud-Kivu Province) (Fig. 1).

#### Comments.

The generated sequence was 658 bp in length and only had 0.2% of ambiguous sites. Using BOLD’s tree-based identification tool, the sequence was sister to all available Pisuliidae sequences on the platform. Additionally, the sequence was most similar to an unidentified male adult from the Eastern Cape Province of South Africa, with an 89.14% similarity. However, the South African sequence was only 621 bp long.

### ﻿ Silvatares laetae

Taxon classificationAnimaliaTrichopteraPisuliidae

Ngirinshuti & Johanson, 2019

5A67BF66-EE74-599C-9410-CF6A9BE57AE3

Silvatares laetae Ngirinshuti & Johanson, 2019 [type locality: Rwanda: Wester Province: Nyamasheke District, Nyungwe National Park, Gisakura, Karamba River; NRS; ♂].

#### Material examined.

Democratic Republic Of The Congo • ♂; Sud-Kivu, Kahuzi-Biega National Park, Tshibati sector, Chashoga swamp; 2.21706°S, 28.7785°E, 2,030 m a.s.l.; 10 Jul. 2005; S. U. Pauls; collected from vegetation using a hand net (SMF). New country record.

#### Comments.

This species has recently been described from the Nyungwe National Park in southwestern Rwanda, and it is one of the few species with broad distributions; however, this is a new distributional record. The male genitalia are identical to the illustrations provided in the original description.

## ﻿Discussion

The Pisuliidae are a group of caddisflies with very interesting biogeography. Almost all species known to date are endemics from a single or very few sites in mountain ranges in Sub-Saharan Africa (Stoltze 1989). There are exceptions, however. For example, *S.crassus* is widespread in the mountains of South-Eastern Africa (Stoltze 1989), and *S.laetae* occurs in Rwanda and the eastern Democratic Republic of the Congo. In addition to this biogeographic pattern, their diversity is likely underestimated. For example, Gibon et al. (2001) estimated more than 20 still undescribed species in Madagascar.

Although *Silvatares* larvae often occur in large numbers, most species are known from very few adults (Stoltze 1989). For example, our new species is only known from a single specimen, as is the new country record of *S.laetae.* This might indicate that species of *Silvatares* are not crepuscular but active during the day, and using a combination of collecting methods such as larval collections with subsequent adult/larval associations (e.g., Graf et al. 2005), Malaise traps and UV pan traps would be more appropriate to estimate their diversity and abundance better. *Silvatareslaetae* from Chashoga swamp was, for example, also collected by day sweeping.

The new species *Silvataresholzenthali*, along with *S.excelsus*, *S.furcifer*, and *S.laetae*, is the fourth species of *Silvatares* recorded from the Democratic Republic of the Congo. Both species treated in this paper were collected in the Tshibati sector in the Kahuzi-Biega National Park. These species belong to the *thrymmifer* group, which is characterized by a pair of apical spines on the endotheca. While knowledge on their distributions is limited, it is interesting to note that the *thrymmifer* group is known from East and South Africa, while the *ensifer* group is known from West and South Africa. *Silvataresfurcifer* is only known from females collected near the type locality of *S.excelsus*, and Prather and Holzenthal (2002) hypothesized that it is conspecific with *S.excelsus*, potentially reducing the number of known species from the D. R. of the Congo to three. However, the presence of additional potential sites for their occurrence (especially in central and south DRC), the distributional pattern of most species in the genus, and their daily activity patterns, additional undescribed *Silvatares* species might occur in the country.

Kahuzi-Biega National Park is listed as a threatened world heritage site, particularly for its high levels of biodiversity associated with the vast mountain and lowland rainforests. This status is based on the better known mammal, bird, and plant diversity. In contrast very little is known about the status of the insect fauna (e.g., Ngera et al. 2019). Considering that we know of only three (or four) species of the rare genus Silvatares in a very small section of the National Park suggests that more extensive surveys will likely reveal great caddisfly species diversity in these old rainforest habitats.

## Supplementary Material

XML Treatment for Silvatares holzenthali

XML Treatment for Silvatares laetae
